# A novel clinical FIT model predicts prognosis of unresectable hepatocellular carcinoma treated with TACE combined with lenvatinib and PD-1 inhibitors therapy

**DOI:** 10.3389/fimmu.2026.1906593

**Published:** 2026-09-22

**Authors:** Minjun Liao, Zilong Wang, Xiaoxiao Wang, Xin Ai, Linnan Song, Yuyun Song, Wanying Qin, Kai Wang, Yuchen Zhou, Feng Liu, Huiying Rao

**Affiliations:** 1 Peking University People’s Hospital, Peking University Hepatology Institute, Infectious Disease and Hepatology Center of Peking University People’s Hospital, Beijing Key Laboratory of Innovative Drug and Device Translation in Endocrine and Metabolic Diseases, Beijing, China; 2 Department of Gastroenterology, Nanfang Hospital, Southern Medical University, Guangzhou, Guangdong, China; 3 Department of Infectious Diseases, Nanfang Hospital, Southern Medical University, Guangzhou, Guangdong, China; 4 Laboratory of Hepatobiliary and Pancreatic Surgery, The First Affiliated Hospital of Guilin Medical University, Guilin, Guangxi, China; 5 Department of General Surgery, Division of Hepatobiliopancreatic Surgery, Nanfang Hospital, Southern Medical University, Guangzhou, Guangdong, China; 6 Guangdong Provincial Key Laboratory of Gastroenterology, Department of Gastroenterology and Hepatology Unit, Nanfang Hospital, Southern Medical University, Guangzhou, Guangdong, China; 7 Department of General Surgery, Integrated Hospital of Traditional Chinese Medicine, Southern Medical University, Guangzhou, Guangdong, China

**Keywords:** fit model, hepatocellular carcinoma, lenvatinib, PD-1, TACE

## Abstract

**Background:**

Transcatheter arterial chemoembolization (TACE) with lenvatinib and programmed death-1 (PD-1) inhibitors therapy (triple therapy) demonstrated encouraging efficacy for unresectable hepatocellular carcinoma (uHCC). This study developed and validated a novel prognostic model to evaluate prognosis of uHCC patients with triple therapy, and identify patients who may benefit from triple therapy.

**Methods:**

uHCC patients receiving triple therapy were retrospectively enrolled from multiple centers. Overall survival (OS) was the primary endpoint. The prognostic model was developed based on multivariate Cox regression derived predictors of OS. Prognosis was evaluated by Kaplan-Meier survival curves.

**Results:**

265 uHCC patients were enrolled (training cohort: n = 160; validation cohort: n = 105). The objective response and disease control rate were 52.5 and 77.7%, respectively. The optimal cut-off point for fibrin degradation products (FDP) and serum iron level were determined as 4.7 μg/dL and 60.0 μg/dL, respectively. Multivariate Cox regression identified FDP, serum iron level and tumor burden score (TBS) as independent predictors of OS, and these clinical parameters established the FIT model. The FIT model demonstrated favorable discriminative capability for predicting OS with AUC of 0.809 (95% CI: 0.740-0.879) and 0.798 (95% CI: 0.710-0.886) in the training and validation cohorts, respectively. Kaplan-Meier analyses revealed significant prognostic stratification for OS and progression-free survival (PFS) among risk groups defined by FIT model (*p* < 0.001). Subgroup analyses in alpha-fetoprotein (AFP), tumor number and Barcelona Clinic Liver Cancer (BCLC) stage subsets confirmed the prognostic relevance of FIT model in uHCC.

**Conclusion:**

The novel clinical FIT model could effectively predict survival of uHCC patients receiving triple therapy, and facilitate prognostic stratification.

## 1 Introduction

Hepatocellular carcinoma (HCC) is the sixth most prevalent and third leading cause of cancer-related death worldwide, which is characterized by latent onset and high malignancy. Treatments for HCC depend on tumor stage and hepatic function, however, more than half of patients are diagnosed as unresectable HCC (uHCC) and not candidates for radical resection (1).

Substantial progression has been made as for therapeutic approaches for uHCC, which includes localized as well as systematic treatments. Transcatheter arterial chemoembolization (TACE) is a well-established therapeutic method for advanced HCC, by delivering chemotherapeutic drugs and obstructing feeding vessels of tumors, which is widely employed in clinical practice (2, 3). Lenvatinib, a tyrosine kinase inhibitor, is approved as a first-line targeted therapy for uHCC according to the clinical trials’ results (4–6). And the use of immune checkpoint inhibitors (ICIs) also demonstrates promising results for advanced HCC patients. To further reduce tumor burden and improve survival for uHCC, the combination regimens are introduced, and the combination of TACE with lenvatinib and PD-1/PD-L1 inhibitors demonstrates favorable efficacy and survival benefit among advanced HCC (7–12). Although the triple therapy has demonstrated encouraging therapeutic efficacy in clinical practice, there is significant individual heterogeneity in treatment response, and several patients experience disease progression and some patients suffer from drug-related adverse reactions. Therefore, prediction of prognostic risk and evaluation of triple therapy efficacy and resistance remain as research tasks at the moment.

Previous studies developed scoring models to predict prognosis and survival of uHCC patients receiving TACE-lenvatinib-PD-1 inhibitors therapy. Recently, Zeng et al. established the TAE score (13), with three variables (total bilirubin, alpha-fetoprotein, and extrahepatic metastasis) being included. While Deng et al. (14) established the EAPTT model, and the Eastern Cooperative Oncology Group performance status (ECOG-PS), albumin-bilirubin grade, platelet-to-lymphocyte ratio, tumor distribution, and total bilirubin were identified as independent predictors of objective response. However, the variables included in the established models were various, and it was unclear which variables were more crucial and significant currently. In this study, we developed and validated a FIT prognostic scoring model based on fibrin degradation products (FDP), serum iron, and tumor burden score (TBS). The FIT scoring model could guide and optimize patient recruitment and provide a reference for triple therapy efficacy evaluation of the TACE-lenvatinib-PD-1 inhibitors regimen.

## 2 Methods

### 2.1 Patient enrollment

HCC was diagnosed based on pathological or clinical-radiological characteristics according to the criteria of the American Association for the Study of Liver Diseases (15). Unresectability of tumor was determined by the multidisciplinary team (MDT). A total of 265 patients diagnosed with uHCC who received triple therapy at 3 medical centers were included in the retrospective study, from December 2018 to June 2022. Patients from Peking University People’s Hospital and Nanfang Hospital of Southern Medical University were grouped in the training cohort, and patients from the First Affiliated Hospital of Guilin Medical University were grouped in an independent external validation cohort. The study was conducted in accordance with the principles of the Declaration of Helsinki and approved by the institutional review board of each center. Written informed consent was obtained from all participants before the initiation of combined therapy.

Patients were enrolled according to the inclusion and exclusion criteria (**Figure 1**). The inclusion criteria were: (1) Age > 18 years; (2) Eastern Cooperative Oncology Group performance status (ECOG-PS) score 0-1; (3) uHCC patients receiving triple therapy (TACE + lenvatinib + PD-1 inhibitors) as first-line treatment; (4) At least one measurable tumor lesion satisfied the modified Response Evaluation Criteria in Solid Tumors (mRECIST) criteria (16). The exclusion criteria were: (1) Child-Pugh C stage; (2) Patients previously received local or systematic treatment; (3) Combination with other malignancies; (4) Patients suffering from autoimmune diseases or immune deficiency; (5) Incomplete clinical data or loss to follow-up.

**Figure 1:**
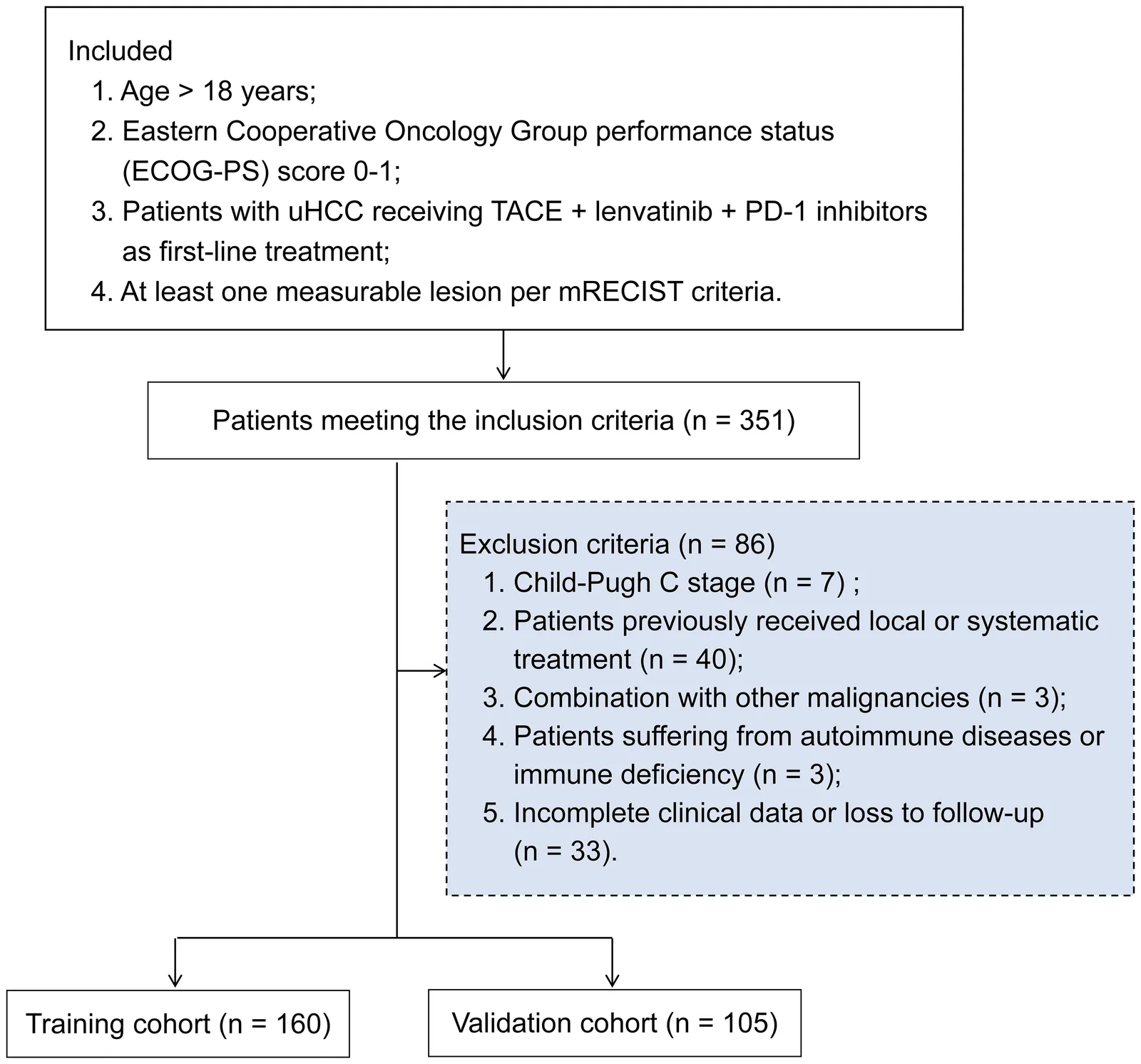
uHCC patients’ enrollment diagram.

### 2.2 Triple therapy regimen

Each patient in this study received triple therapy (TACE + lenvatinib + PD-1 inhibitors). TACE was performed by interventional specialists with more than 5 years of experiences. Tumor characteristics and vascular anatomical structure were determined through angiography by selective intubation performed on the celiac trunk and hepatic artery. Subsequently, the mixed emulsion of iodized oil (5–20 mL) and chemotherapy drugs was injected into the tumor supplying arteries. Finally, gelatin sponge granules were used to embolize the supplying artery. The dosage of iodized oil (≤ 20 mL per time) and chemotherapy drugs were adjusted individually. TACE was repeated every 4 to 6 weeks depending on intrahepatic viable or progressive tumor with acceptable liver functional reserve, and discontinued if (1) liver function deteriorated to Child-Pugh C stage (2); ECOG-PS > 1 (3); target lesions progressed after three TACE procedure (4); no treatable arteries available.

Lenvatinib and PD-1 inhibitors were initiated within 3 to 14 days after the initial TACE procedure after assessment of patient’s general condition and hepatic function recovery. Lenvatinib was treated orally (body weight < 60 kg: 8 mg/day; body weight ≥ 60 kg: 12 mg/day). PD-1 inhibitors were administered intravenously (sintilimab, camrelizumab, pembrolizumab or tislelizumab, 200 mg once, every 3 weeks). Lenvatinib and PD-1 inhibitors should be discontinued for 3 days before and after TACE procedure. Therapy continued until disease progression or intolerable toxicity.

### 2.3 Evaluation of triple therapy efficacy and safety

The treatment responses were determined according to the modified Response Evaluation Criteria in Solid Tumors (mRECIST) (17), and classified into complete response (CR), partial response (PR), stable disease (SD), and progressive disease (PD). The primary endpoint was overall survival (OS) in the current study, defined as the time interval from initial treatment to occurrence of death from any cause or end of follow-up. Secondary endpoints included progression free survival (PFS), objective response rate (ORR), disease control rate (DCR) and treatment-related adverse events (TRAEs). PFS was defined as the time from treatment initiation until disease progression or death from any cause. ORR was defined as the proportion of CR or PR, sustained for at least 4 weeks. DCR was defined as the proportion of CR, PR, and SD. Drug safety was evaluated by the incidence and severity of TRAEs, which was monitored and assessed according to the Common Terminology Criteria for Adverse Events (CTCAE).

### 2.4 Follow-up surveillance

All patients were routinely followed up and monitored through laboratory tests and radiological examinations by contrast-enhanced CT or MRI every 4 to 8 weeks, and tumor resectability was evaluated at each follow up. Lenvatinib and PD-1 inhibitors should be suspended for one and four weeks before and after surgery, respectively. Postoperative lenvatinib and PD-1 inhibitors were continued for 3 to 12 weeks. On other cases, patients continued the triple therapy until disease progression, unacceptable toxicity or treatment refusal.

### 2.5 Statistical analysis

Statistical analyses were conducted using IBM SPSS Statistics software (version 26.0, IBM Corp., Armonk, NY, USA) and MedCalc software (MedCalc Software Ltd., Ostend, Belgium). Continuous variables were presented as mean ± standard deviation (SD) or median with interquartile range (IQR), and compared using the Student’s t-test or Mann-Whitney U test. Categorical variables were presented as frequencies with percentages and analyzed with Pearson’s Chi-Square Test (χ^2^ test) or Wilcoxon rank sum test. Survival curves were analyzed using Kaplan-Meier method and compared with log-rank test. In training cohort, prognostic factors for OS were determined using univariate and multivariate Cox regression. And in training cohort, the receiver operating characteristic (ROC) curve was plotted using OS as the outcome to determine the optimal cutoff value for continuous variables, and the Youden index was used to select the threshold with the optimal combination of sensitivity and specificity. The predictive ability of model was evaluated by ROC curves with area under the curve (AUC) quantification. The prognostic scoring model was validated using an independent external validation cohort. A two-sided *p* value < 0.05 was considered statistically significant.

## 3 Results

### 3.1 Patient baseline characteristics

The baseline characteristics of 265 uHCC (training: 160; validation: 105) receiving triple therapy were shown in **Table 1**. Baseline characteristics were comparable between two cohorts, with no statistical difference (*p* > 0.05). Among all patients, male patients accounted for the majority population (85.3%), the average age was 57.02 years, viral hepatitis being the predominant etiology (83.0%), 172 patients had multiple tumors (64.9%), 58 patients had extrahepatic metastasis (21.9%), 141 patients had portal vein tumor thrombus (53.2%), and 167 patients were Barcelona Clinic Liver Cancer (BCLC)-C stage (63.0%). In this study, 38 patients satisfied the surgical criteria and received resection (21 in the training cohort and 17 in the validation cohort).

**Table 1:** Comparison of clinicopathological characteristics of two cohorts’ patients.

Parameter	Total patients (n = 265)	Training cohort (n = 160)	Validation cohort (n = 105)	*p* value
Age (years)	57.02±11.39	56.35±11.74	58.03±10.83	0.557
Gender: female/male (n)	39/226	28/132	11/94	0.114
BMI	22.05±3.05	21.86±2.89	22.34±3.27	0.278
Alcohol abuse: no/yes (n)	163/102	102/58	61/44	0.354
Smoking: no/yes (n)	186/79	118/42	68/37	0.118
HBsAg: negative/positive (n)	45/220	28/132	17/88	0.781
WBC (×10^9^/L)	6.55±2.34	6.58±2.42	6.50±2.24	0.248
LYMPH (×10^9^/L)	1.44±0.60	1.44±0.64	1.45±0.54	0.192
Platelets (×10^9^/L)	190.59±100.53	196.79±100.71	181.14±100.01	0.983
PT (sec)	12.89±2.52	13.07±3.01	12.62±1.47	0.092
APTT (sec)	31.00±5.75	31.04±6.10	30.82±4.95	0.157
INR	1.12±0.23	1.13±0.27	1.10±0.14	0.167
TT (sec)	18.65±2.62	18.94±2.58	18.26±2.71	0.055
FIB (g/L)	3.59±2.14	3.77±2.68	3.31±1.28	0.088
FDP (μg/dL)	5.11±4.05	5.34±4.33	4.75±3.55	0.220
Albumin (g/L)	35.44±5.65	35.66±5.95	35.09±5.18	0.089
Globulin (g/L)	36.53±7.73	37.01±7.68	35.78±7.77	0.914
TBIL (μmol/L)	17.95±11.71	17.92±12.57	18.00±10.31	0.089
DBIL (μmol/L)	10.03±8.76	10.28±9.50	9.64±7.53	0.112
ALT (U/L, median, IQR)	33.06 (21.15-51.95)	30.85 (20.40-51.45)	35.80 (24.90-52.05)	0.274
AST (U/L, median, IQR)	50.30 (31.55-87.60)	48.85 (31.32-93.52)	50.90 (35.17-78.1)	0.101
GGT (U/L, median, IQR)	120.0 (79.5-211.76)	120.0 (79.25-205.25)	114.0 (79.27-214.8)	0.119
ALP (U/L)	152.52±97.44	159.89±109.48	141.30±74.62	0.077
AFP (ng/mL): ≤ 400/> 400	140/125	83/77	57/48	0.701
Iron (μg/dL)	78.81±42.67	78.41±40.84	80.50±44.65	0.174
ECOG-PS (0/1)	166/99	101/59	65/40	0.841
Tumor size (cm)	9.87±3.94	10.35±3.95	9.13±3.82	0.978
TNb: single/multiple (n)	93/172	61/99	32/73	0.202
TBS	8.65±3.66	9.03±3.69	8.14±3.63	0.962
EHS: no/yes (n)	207/58	122/38	85/20	0.365
PVTT: no/yes (n)	124/141	75/85	49/56	0.973
Child-Pugh: A/B (n)	193/72	116/44	77/28	0.881
BCLC stage: B/C (n)	98/167	57/103	41/64	0.572
CS: no/yes (n)	227/38	139/21	88/17	0.486

n, number of patients; BMI, body mass index; HBsAg, hepatitis B surface antigen; WBC, white blood cell; LYMPH, lymphocyte count; PT, prothrombin time; APTT, activated partial coagulation time; INR, international normalized ratio; TT, thrombin time; FIB, fibrinogen; FDP, fibrin degradation products; TBIL, total bilirubin; DBIL, direct bilirubin; ALT, alanine aminotransferase; IQR, Interquartile range; AST, aspartate aminotransferase; GGT, Gamma-glutamyl transpeptidase; ALP, alkaline phosphatase; AFP, alpha-fetoprotein; ECOG-PS, eastern cooperative oncology group performance status; TNb, tumor number; TBS, tumor burden score; EHS, extrahepatic spread; PVTT, portal vein tumor thrombus; BCLC, Barcelona Clinic Liver Cancer; CS, conversion surgery.

### 3.2 Treatment response and safety assessment

Based on the mRECIST criteria, CR was observed in 56 (21.1%), PR in 83 (31.3%), SD in 67 (25.3%), and PD in 59 (22.3%) patients. The ORR were 51.3% and 54.3%, while DCR were 75.6% and 80.9% in the training and validation cohorts, respectively (**Table 2**).

**Table 2:** Radiological response according to mRECIST in the training and validation cohorts.

Variable	Total patients (n = 265, %)	Training cohort (n = 160, %)	Validation cohort (n = 105, %)
Complete response	56 (21.1)	35 (21.9)	21 (20.0)
Partial response	83 (31.3)	47 (29.4)	36 (34.3)
Stable disease	67 (25.3)	39 (24.4)	28 (26.7)
Progressive disease	59 (22.3)	39 (24.4)	20 (19.0)
Objective response rate	139 (52.5)	82 (51.3)	57 (54.3)
Disease control rate	206 (77.7)	121 (75.6)	85 (80.9)

n, number of patients; mRECIST, modified response evaluation criteria in solid tumors.

Among all uHCC patients receiving triple therapy, TRAEs were frequent but largely controllable (**Supplementary Table 1**). In training cohort, 124 (77.5%) patients experienced any grade TRAEs, among which 96 (60.0%) were Grade 1-2, 24 (15.0%) were Grade 3, and 4 (2.5%) were Grade 4. Similarly, 77 (73.3%) patients reported any grade TRAEs, including 57 (54.3%) Grade 1-2, 17 (16.2%) Grade 3, and 3 (2.9%) Grade 4 in the validation cohort. The most common TRAEs for both cohorts were abnormal liver function (57.5% in training, 64.8% in validation), fever (29.4% in training, 30.5% in validation), and nausea (23.8% in training, 26.7% in validation). No treatment-related deaths occurred, and most moderate to severe events were controlled through drug dose adjustment or temporary withdrawal.

### 3.3 Determination of independent predictors and FIT prognostic model

In training cohort, the univariate Cox regression was used to evaluate relationship between continuous variables and OS. The results showed that FDP (*p* < 0.001), serum iron (*p* = 0.001) and TBS (*p* = 0.013) were associated with OS. Therefore, to enhance clinical interpretability and facilitate the construction of a simple scoring system, the continuous variables were treated as binary variables. The optimal cut-off value of fibrin degradation products (FDP) for predicting OS was 4.7 μg/dL, with an AUC of 0.719 (95% confidence interval (95% CI): 0.639-0.799, sensitivity: 72.3%, specificity: 70.1%); and the optimal cut-off value of Iron was 60.0 μg/dL, with an AUC of 0.703 (95% CI: 0.623-0.783, sensitivity: 62.5%, specificity: 71.9%), according to the results of the ROC curves (**Figures 2A, B**). Tumor size was measured in centimeters (cm); and the formula of TBS was: TBS^2^ = (maximum tumor diameter)^2^ + (number of tumors)^2^, with cutoff value of TBS determined as 8.0 (18).

**Figure 2:**
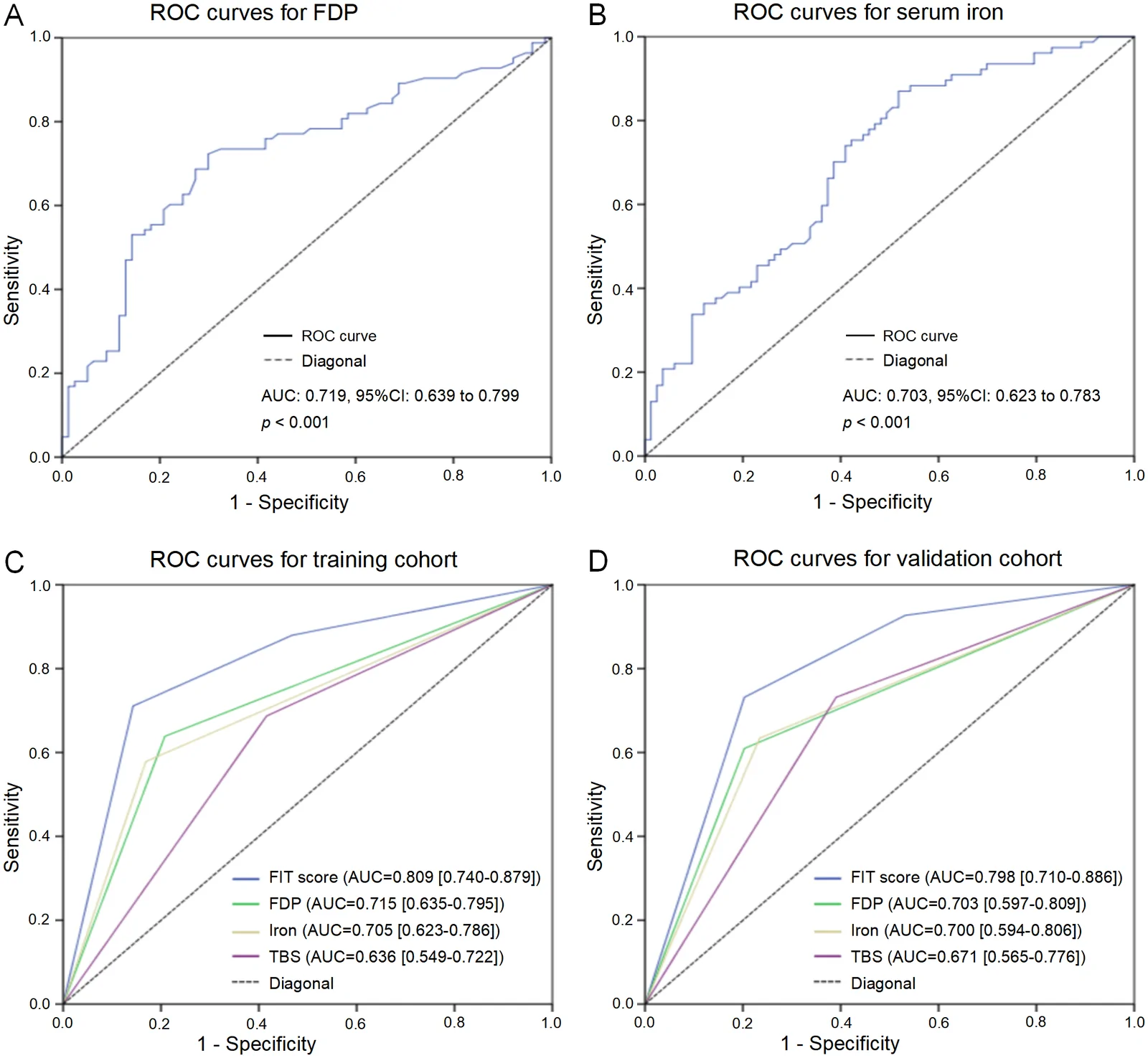
Determination of optimal cut-off value of FDP **(A)** and Iron **(B)** for predicting OS. The predictive capability of FIT scoring model based on ROC curve analysis. AUC of the FIT model, FDP, Iron, and TBS in training cohort **(C)**, and validation cohort **(D)**.

After processed as binary variables, in univariate Cox regression analysis, FDP (*p* < 0.001), alpha-fetoprotein (AFP) (*p* = 0.02), Iron (*p* < 0.001), tumor burden score (TBS) (*p* = 0.001) and portal vein tumor thrombus (PVTT) (*p* = 0.013) were associated with poor OS (**Table 3**). Multivariate Cox regression suggested FDP (HR = 1.802; 95% CI: 1.136-2.861; *p* = 0.012), Iron (HR = 1.747; 95% CI: 1.083-2.817; *p* = 0.022), and TBS (HR = 1.663; 95% CI: 1.003-2.759; *p* = 0.048) as independent prognostic predictors of OS (**Table 3**).

**Table 3:** Univariate and multivariable Cox regression analyses of OS in the training cohort.

	Univariate		Multivariate		
Variable	HR (95% CI)	*p* value	β-estimate	HR (95% CI)	*p* value
Age, years (> 55 *vs.* ≤ 55)	0.705 (0.454-1.093)	0.118			
Gender (male *vs.* female)	0.863 (0.484-1.538)	0.618			
BMI (> 24 *vs*. ≤ 24)	0.592 (0.293-1.195)	0.143			
Alcohol abuse (yes *vs*. no)	0.774 (0.435-1.379)	0.385			
Smoking (yes *vs*. no)	0.808 (0.473-1.381)	0.435			
HBsAg (positive *vs*. negative)	0.711 (0.415-1.216)	0.212			
FDP, μg/dL (> 4.7 *vs.* ≤ 4.7)	2.398 (1.543-3.729)	< 0.001	0.589	1.802 (1.136-2.861)	0.012
ALT, U/L (> 38.0 *vs*. ≤ 38.0)	1.298 (0.833-2.022)	0.250			
AFP, ng/mL (> 400 *vs*. ≤ 400)	1.696 (1.088-2.644)	0.020		1.212 (0.761-1.929)	0.418
Iron, μg/dL (≤ 60.0 *vs.* >60.0)	2.298 (1.478-3.571)	< 0.001	0.558	1.747 (1.083-2.817)	0.022
ECOG-PS (1 *vs*. 0)	1.150 (0.734-1.804)	0.542			
Tumor size, cm (> 5 *vs*. ≤ 5)	2.168 (0.810-6.141)	0.109			
TNb (multiple *vs*. single)	1.191 (0.752-1.886)	0.457			
TBS (> 8.0 *vs*. ≤ 8.0)	2.177 (1.368-3.464)	0.001	0.509	1.663 (1.003-2.759)	0.048
PVTT (yes *vs*. no)	1.817 (1.137-2.903)	0.013		1.149 (0.680-1.940)	0.604

HR, hazard ratio; CI, confidence interval; HBsAg, hepatitis B surface antigen; FDP, fibrin degradation products; ALT, alanine aminotransferase; AFP, alpha-fetoprotein; ECOG-PS, eastern cooperative oncology group performance status; TNb, tumor number; TBS, tumor burden score; PVTT, portal vein tumor thrombus.

The prognostic prediction model was determined as FIT score, which was composed of FDP, Iron and TBS. And FIT model was calculated as: FIT score = 0.589 × FDP (μg/dL) (0: ≤ 4.7; 1: > 4.7) + 0.558 × Iron (μg/dL) (0: > 60.0; 1: ≤ 60.0) + 0.509 × TBS (0: ≤ 8.0; 1: > 8.0).

### 3.4 The performance of FIT prognostic model

The predictive efficacy of the novel FIT model was evaluated by the AUC quantification. The FIT scoring model demonstrated favorable discriminative performance in both training and validation cohorts in predicting OS of uHCC patients who received triple therapy. As shown in **Figure 2**, the FIT model showed higher AUC values compared with other single variables in the training cohort (AUC = 0.809, 95% CI: 0.740-0.879; FDP: 0.715, Iron: 0.705, TBS: 0.636) (**Figure 2C**), and either comparable discrimination and robustness within the validation cohort (AUC = 0.798, 95% CI: 0.710-0.886; FDP: 0.703, Iron: 0.700, TBS: 0.671) (**Figure 2D**).

Moreover, we conducted comparative analyses with ALBI, BCLC stage, AFP and TAE scores, and found that FIT score outperformed other models or scores in terms of predictive performance, with AUC of 0.809 (*vs*. 0.626 for ALBI, 0.663 for BCLC stage, 0.592 for AFP, and 0.708 for TAE) in the training cohort and 0.798 (*vs*. 0.630 for ALBI, 0.680 for BCLC stage, 0.569 for AFP, and 0.701 for TAE) in the validation cohort (**Supplementary Figure 1**).

### 3.5 Survival analysis

In training cohort, the median OS was 19.4 months (95% CI: not reached-not reached), and median PFS was 12.0 months (95% CI: 9.4-14.6), as demonstrated by Kaplan-Meier survival. In validation cohort, the median OS was not reached, and median PFS was 11.0 months (95% CI: 6.2-15.7) (**Supplementary Figure 2**).

The FIT model could stratify uHCC patients into three subgroups, and patients showed different survival after triple therapy, which included low (FIT score ≤ 0.5), intermediate (0.5 < FIT score ≤ 1.1) and high-risk subgroups (FIT score > 1.1) (**Figure 3**). Kaplan-Meier survival suggested significant differences in terms of OS among those uHCC patients classified into low, intermediate, and high FIT scoring groups in both training and validation cohorts (**Figures 3A, C**). And log-rank tests further confirmed the differences with *p* < 0.001. Moreover, PFS of different risk groups were either analyzed, with higher FIT scoring owing the shortest PFS (**Figures 3B, D**). These results collectively suggested that the current FIT scoring model could effectively stratify uHCC patients by survival risk and may serve as a reliable predictor of therapy efficacy for triple therapy in uHCC.

**Figure 3:**
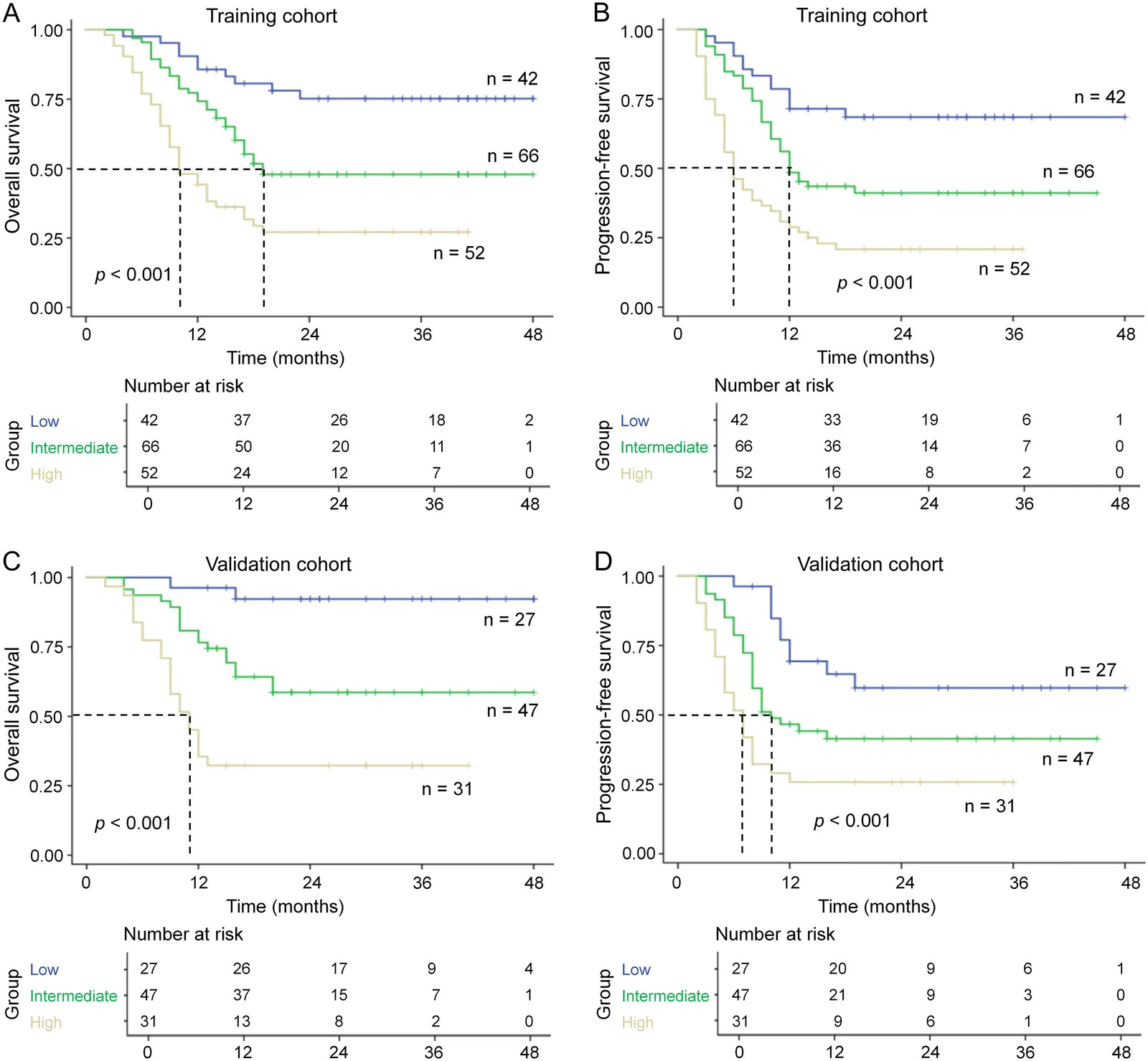
Kaplan-Meier survival curve analyses based on the FIT scoring model. uHCC patients were divided into three subgroups with different survival risks based on the FIT scoring model, including low-risk (FIT score ≤ 0.5), intermediate-risk (0.5 < FIT score ≤ 1.1), and high-risk group (FIT score > 1.1). OS and PFS in training cohort **(A, B)** and validation cohort **(C, D)**.

In the current study, the FIT score was further analyzed for its prognostic predictive effect in subgroups of key clinical variables. In the training cohort, the FIT score demonstrated favorable predictive capability in various subgroups, including AFP (≤ 400 ng/ml, > 400 ng/ml), tumor number (single, multiple), and BCLC stage (B, C) (**Figure 4**). In the validation cohort, heterogeneity was observed in the subgroup of BCLC-B stage (log-rank tests, OS: *p* = 0.074, PFS: *p* = 0.089) (**Supplementary Figure 3**), while the FIT score remained predictive effects in other subgroups. The inconsistent result observed in the BCLC-B subgroup, might be partly resulted from limited sample size, with 41 patients in the BCLC-B subgroup in validation cohort, and 26, 11 and 4 patients in the low-, intermediate- and high-risk groups defined by FIT, respectively, suggesting that the FIT model may not be robust enough in less advanced stages.

**Figure 4:**
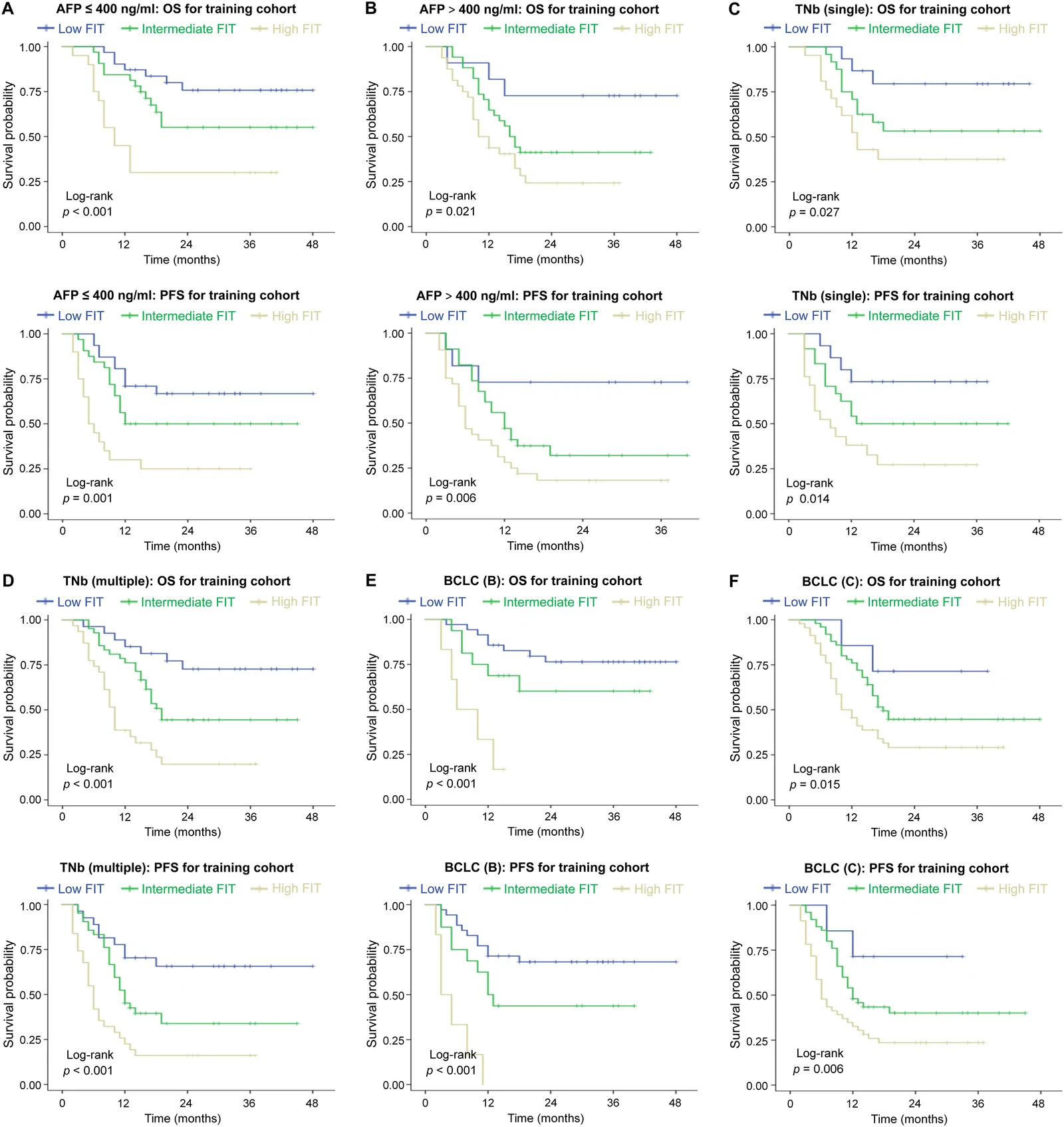
Subgroup analyses of survival outcomes stratified by clinical characteristics in the training cohort. **(A)** OS and PFS of patients with AFP ≤ 400 ng/mL (log-rank, OS: *p* < 0.001, PFS: *p* = 0.001); **(B)** AFP > 400 ng/mL (log-rank, OS: *p* = 0.021, PFS: *p* = 0.006); **(C)** single tumor (log-rank, OS: *p* = 0.027, PFS: *p* = 0.014); **(D)** multiple tumors (log-rank, OS: *p* < 0.001, PFS: *p* < 0.001); **(E)** BCLC-B stage (log-rank, OS: *p* < 0.001, PFS: *p* < 0.001); **(F)** BCLC-C stage (log-rank, OS: *p* = 0.015, PFS: *p* = 0.006). OS, overall survival; PFS, progression free survival; AFP, alpha-fetoprotein; TNb, tumor number; BCLC, Barcelona Clinic Liver Cancer.

## 4 Discussion

In many clinical trial studies, systematic therapy regimen was recommended for advanced patients, among which, TACE combined with lenvatinib and PD-1 inhibitors therapy demonstrated inspiring and significant efficacy in uHCC (8, 12, 19). The triple therapy significantly enhanced anti-tumor efficacy through synergistic mechanisms (20). Partially, TACE realized local arterial occlusion and cytotoxic killing to achieve tumor reduction. Moreover, TACE operation led to stimulation of vascular endothelial growth factor (VEGF) and fibroblast growth factor (FGF) and release of tumor associated antigens, which formed foundation of complement of lenvatinib with TACE, a multitarget kinase inhibitor against VEGF and FGF receptors and alleviating immunosuppression, to collectively induce tumor necrosis and inhibition (12). Furthermore, implementation of PD-1 inhibitors permitted T-cell infiltration and function (21), functioning as supplementary strike against the residual tumor cells, which may partly explain the delay of aggressive tumor features, including microvascular invasion (MVI) and extrahepatic spread (EHS) after the addition of PD-1 inhibitors (22).

A retrospective cohort study among huge uHCC (maximum diameter ≥ 10 cm) demonstrated TACE + lenvatinib + PD-1 inhibitor with higher ORR and prolonged OS and PFS, and these beneficial improvements may be demonstrated by early tumor response and control achieved by triple regimen (23). Despite of the inspiring outcomes, approximately 30% of the uHCC patients failed to respond to combined triple therapy and drug resistance remained as clinical challenges, which may be partly explained by tumor heterogeneity and individual differences (14, 24). Additionally, triple regimen associated with increasing incidence of TRAEs, which may adversely affect patient survival. Therefore, identification of appropriate participants and accurate stratification for triple therapy were essential. This study determined and validated the FIT model, which was a novel prognostic model incorporating three clinical factors (FDP, Iron and TBS) to predict treatment response and survival outcomes of uHCC patients who received TACE plus lenvatinib and PD-1 inhibitors. The FIT scoring demonstrated favorable discrimination in patient stratification, as well as significant relevance with patient’s prognosis.

Elevated FDP level was a key biomarker of coagulation-fibrinolysis activation. Fibrinogen, fibrin and their degradation products had proinflammatory activity, and may contribute to tumor vascular remodeling (25), meanwhile, FDP may bind to immune cell related receptors, activate inflammatory signaling pathways, release inflammatory factors, promote local inflammatory microenvironment in tumor, and suppress anti-tumor immunity. At the same time, FDP might induce endothelial cell proliferation, upregulate pro-angiogenic factors, degrade extracellular matrix, increase vascular permeability and therefore promote tumor growth and metastasis. Previous studies demonstrated correlations of elevated FDP level with HCC and other malignancies, as well as with liver dysfunction (26). And a correlation analysis pointed out higher FDP and D-dimer levels in the AFP-positive HCC compared with AFP-negative HCC (27). In the current study, we also found that FDP may contribute to risk stratification and was relevant with treatment response.

In our previous study, preoperative serum low iron level was an unfavorable independent predictor of HCC, which correlated with survival and recurrence after resection (28). Another study also pointed out that serum iron level < 15.1 μmol/L was relevant with poor prognosis of HBV-related HCC (29). However, Yu. et al. found that elevated serum iron level may increase HCC risk among NAFLD patients (30). Hepcidin positively altered with body iron reserve and thereafter negatively regulated the absorption of iron. And hepcidin, a key hormone regulating iron metabolism and maintaining systematic iron homoeostasis, was either involved in HCC pathology (31). And generally, decrease of serum iron may reflect an imbalance of iron metabolism throughout the body and tumor, hepcidin-mediated iron regulation, oxidative stress alteration, and vulnerability related to ferroptosis. Serum iron had potential effects on tumor immune microenvironment under PD-1 blockade treatment conditions, as ferroptosis and iron metabolism may interact with antigen release, immune cell metabolism, and effector T cell function. These results may put forward the foundation of underlying significance of serum iron level in HCC.

TBS score was regarded as a survival predictor and prognostic biomarker for HCC (32, 33), and elevated TBS positively correlated with more aggressive tumor biology and adverse outcomes (34). In the retrospective study by Xue. et.al, TBS was also identified as independent prognostic factors for PFS (HR = 1.772, *p* = 0.010) and OS (HR = 1.904, *p* = 0.023) after TACE plus lenvatinib and PD-1 inhibitors regimen (22).

Briefly, abnormal fibrinolysis activation and imbalanced iron metabolism manifested the remodeling of tumor microenvironment and organic adaption to tumor condition. With combination of tumor burden evaluation, the FIT model could provide more comprehensive assessment of systemic treatment response and prognosis discrimination when receiving triple therapy.

The FIT scoring system may attract significant attention from researchers for the advantages of being easily accessible, having good repeatability and being cheap and highly cost-effective. In comparison, the predictive ability of a single variable was limited, which was also confirmed in this study, and in both training and validation cohort, with the AUC value of the FIT score’s efficacy higher than that of the established single variable (FDP, Iron and TBS). To further evaluate the OS predictive performance of FIT score, we included comparative analyses with ALBI, BCLC stage, AFP and TAE scores, and revealed that the FIT score outperformed other models or scores in the two cohorts. These results indicated that the FIT score had significant advantages, as an individualized prognostic tool for uHCC patients receiving TACE combined with lenvatinib and PD-1 inhibitor therapy. Meanwhile, the FIT score could categorize uHCC patients and subgroups’ patients (based on AFP, tumor number, and BCLC stage) into three stratifications of low, intermediate and high risk, providing new strategies for clinical treatment. In the current study, although the univariate analysis indicated AFP > 400 ng/ml and combination with PVTT as predictors of poor OS, and neither of them were determined as independent predictors in the multivariate analysis. However, this result did not suggest there was no correlation between these factors and OS. AFP was a commonly used and vital biomarker for the diagnosis, treatment efficacy and prognosis monitoring of HCC (35–38). Combination with PVTT limited treatment options, and directly affected TACE efficacy and systemic response to treatment (39, 40), and potential mechanism may be partly explained by the abundance of C5aR+ tumor-associated macrophages (TAMs) in PVTT, and that C5aR+ TAMs contributed to PVTT development and poor prognosis through reshaping the immunosuppressive environment (41).

TRAEs increased with the combination of treatment methods (11). Though systematic therapy demonstrated significant improvements in terms of ORR and survival, the adverse events and patient tolerability should be considered. In our study, hepatic impairment was the most common TRAE, in accordance with other literatures, followed by fever and nausea. Most patients tolerated the therapy, and most adverse events were controlled by dose adjustment and temporary withdrawal; and no patient died of the TRAEs, suggesting the efficacy and safety of the regimen.

The current study had certain limitations. Firstly, the retrospective design may lead to potential selection bias, and the follow-up time was relatively short, and we would extend observation to evaluate long-term survival benefits and delayed drug toxicity. Secondly, this study included multiple PD-1 inhibitors. Although all drugs were PD-1 targeted and administered according to standard protocols, potential differences in pharmacokinetics, efficacy and toxicity could not be completely ruled out. Due to the limited sample size and uneven distribution of different PD-1 inhibitors, it was difficult to conduct drug-specific subgroup analyses. Thirdly, most patients in this study were HBV infected, further studies on different baseline characteristics should be carried out to explore treatment response variations and optimize treatment strategies. Finally, the FIT model did not reach statistical significance in the BCLC-B subgroup stratification, suggesting that FIT model may not be robust enough in less advanced stages, therefore, it was necessary for further studies in a larger BCLC-B cohort. Meanwhile, the clinical utility of the FIT model should be further evaluated in larger prospective cohorts from different centers.

In conclusion, the FIT model serves as a convenient and effective tool for personalized management, which can effectively stratify uHCC patients receiving TACE combined with lenvatinib and PD-1 inhibitors therapy into distinct prognostic groups. Future prospective research is necessary to confirm its clinical utility and generalizability, and this may provide support of clinical strategies for uHCC.

## Data Availability

The datasets used and analyzed during the current study are available from the corresponding author upon reasonable request.

## Supplementary material

Supplementary Figure 1 Comparative analyses of predictive performance of FIT score with ALBI, BCLC stage, AFP and TAE score. ROC curves of FIT score, ALBI, BCLC stage, AFP and TAE score in the training cohort **(A)** and validation cohort **(B)**.

Supplementary Figure 2 Kaplan-Meier survival analyses of OS and PFS in uHCC patients received triple therapy. In training cohort **(A, B)**, median OS was 19.4 months and median PFS was 12.0 months. In validation cohort **(C, D)**, median OS was not reached and median PFS was 11.0 months.

Supplementary Figure 3 Subgroup analyses of survival outcomes stratified by clinical characteristics in the validation cohort. **(A)** OS and PFS of patients with AFP ≤ 400 ng/mL (log-rank, OS: *p* = 0.001, PFS: *p* = 0.040); **(B)** AFP > 400 ng/mL (log-rank, OS: *p* = 0.003, PFS: *p* = 0.017); **(C)** single tumor (log-rank, OS: *p* < 0.001, PFS: *p* = 0.003); **(D)** multiple tumors (log-rank, OS: *p* = 0.001, PFS: *p* = 0.015); **(E)** BCLC-B stage (log-rank, OS: *p* = 0.074, PFS: *p* = 0.089); **(F)** BCLC-C stage (log-rank, OS: *p* = 0.004, PFS: *p* = 0.015). OS, overall survival; PFS, progression free survival; AFP, alpha-fetoprotein; TNb, tumor number; BCLC, Barcelona Clinic Liver Cancer.
