# Supplementary material for: A novel clinical FIT model predicts prognosis of unresectable hepatocellular carcinoma treated with TACE combined with lenvatinib and PD-1 inhibitors therapy
Source: Front Immunol. 2026 Sep 22;17:1906593. doi: 10.3389/fimmu.2026.1906593 (PMC13638581; doi:10.3389/fimmu.2026.1906593)
Supplement: Supplementary file 1 [file Table1.docx]

**Table S1.** Treatment-related adverse events.

|  | **Training cohort (n = 160, %)** | | | | | **Validation cohort (n = 105, %)** | | | | | | | | |  |  |
| --- | --- | --- | --- | --- | --- | --- | --- | --- | --- | --- | --- | --- | --- | --- | --- | --- |
| **Adverse Events** | **Any Grade** | **Grade 1-2** | **Grade 3** | | **Grade 4** | | | **Any Grade** | | **Grade 1-2** | | **Grade 3** | | **Grade 4** | | |
| **Total** | 124 (77.5) | 96 (60.0) | 24 (15.0) | 4 (2.5) | | | 77 (73.3) | | 57 (54.3) | | 17 (16.2) | | 3 (2.9) | | |  |
| Fever | 47 (29.4) | 42 (26.3) | 5 (3.1) | – | | | 32 (30.5) | | 26 (24.8) | | 5 (4.8) | | 1 (1.0) | | |  |
| Abnormal liver  function | 92 (57.5) | 82 (51.3) | 8 (5.0) | 2 (1.3) | | | 68 (64.8) | | 62 (59.0) | | 5 (4.8) | | 1 (1.0) | | |  |
| Nausea | 38 (23.8) | 35 (21.90 | 3 (1.9) | – | | | 28 (26.7) | | 25 (23.8) | | 3 (2.9) | | – | | |  |
| Fatigue | 35 (21.9) | 29 (18.1) | 6 (3.8) | – | | | 20 (19.1) | | 17 (16.2) | | 3 (2.9) | | – | | |  |
| Hypertension | 31 (19.4) | 28 (17.5) | 3 (1.9) | – | | | 25 (23.8) | | 22 (21.0) | | 3 (2.9) | | – | | |  |
| Abdominal pain | 23 (14.4) | 19 (11.9) | 4 (2.5) | – | | | 16 (15.2) | | 15 (14.3) | | 1 (1.0) | | – | | |  |
| Hand-foot syndrome | 20 (12.5) | 17 (10.6) | 2 (1.3) | 1 (0.6) | | | 17 (16.2) | | 14 (13.3) | | 3 (2.9) | | – | | |  |
| Weight loss | 11 (6.9) | 10 (6.3) | 1 (0.6) | – | | | 5 (4.8) | | 4 (3.8) | | 1 (1.0) | | – | | |  |
| Diarrhea | 20 (12.5) | 18 (11.3) | 2 (1.3) | – | | | 11 (10.5) | | 10 (9.5) | | 1 (1.0) | | – | | |  |
| Skin rash | 19 (11.9) | 12 (7.5) | 6 (3.8) | 1 (0.6) | | | 16 (15.2) | | 11 (10.5) | | 4 (3.8) | | 1 (1.0) | | |  |
| Hypothyroidism | 21 (13.1) | 17 (10.6) | 4 (2.5) | – | | | 11 (10.5) | | 9 (8.6) | | 2 (1.9) | | – | | |  |
| Proteinuria | 17 (10.6) | 14 (8.8) | 3 (1.9) | – | | | 9 (8.6) | | 7 (6.7) | | 2 (1.9) | | – | | |  |
| Thrombocytopenia | 18 (11.3) | 16 (10.0) | 2 (1.3) | – | | | 15 (14.3) | | 14 (13.3) | | 1 (1.0) | | – | | |  |
